# Intracellular galectin interactions in health and disease

**DOI:** 10.1007/s00281-024-01010-z

**Published:** 2024-07-11

**Authors:** Ralf Jacob, Lena-Sophie Gorek

**Affiliations:** Department of Cell Biology and Cell Pathology, Philipps University of Marburg, Karl-von-Frisch-Str. 14, D-35043 Marburg, Germany

**Keywords:** Galectin, Protein interaction, Immune cell, Cancer

## Abstract

In the galectin family, a group of lectins is united by their evolutionarily conserved carbohydrate recognition domains. These polypeptides play a role in various cellular processes and are implicated in disease mechanisms such as cancer, fibrosis, infection, and inflammation. Following synthesis in the cytosol, manifold interactions of galectins have been described both extracellularly and intracellularly. Extracellular galectins frequently engage with glycoproteins or glycolipids in a carbohydrate-dependent manner. Intracellularly, galectins bind to non-glycosylated proteins situated in distinct cellular compartments, each with multiple cellular functions. This diversity complicates attempts to form a comprehensive understanding of the role of galectin molecules within the cell. This review enumerates intracellular galectin interaction partners and outlines their involvement in cellular processes. The intricate connections between galectin functions and pathomechanisms are illustrated through discussions of intracellular galectin assemblies in immune and cancer cells. This underscores the imperative need to fully comprehend the interplay of galectins with the cellular machinery and to devise therapeutic strategies aimed at counteracting the establishment of galectin-based disease mechanisms.

## Introduction

The glycome contains important biological information and in this way has a major impact on the cellular function and behaviour [1]. Cell tools for glycome manipulation employ glycosyl transferases, glycosidases and lectins. A β-galactoside binding lectin family are the galectins [2]. They have been found in multicellular organisms and can be detected intra- as well as extracellularly. Numerous functions attributed to galectins revolve around extracellular carbohydrate recognition, targeting either glycosylated membrane components or soluble ligands. However, it is noteworthy that galectins are synthesized in the cytosol, a cellular compartment where they can engage in specific interactions with various binding partners. In this intracellular environment, galectins establish connections with cellular entities, showcasing their versatility in influencing processes beyond the realm of extracellular carbohydrate recognition.

## The galectin family

**Fig. 1:**
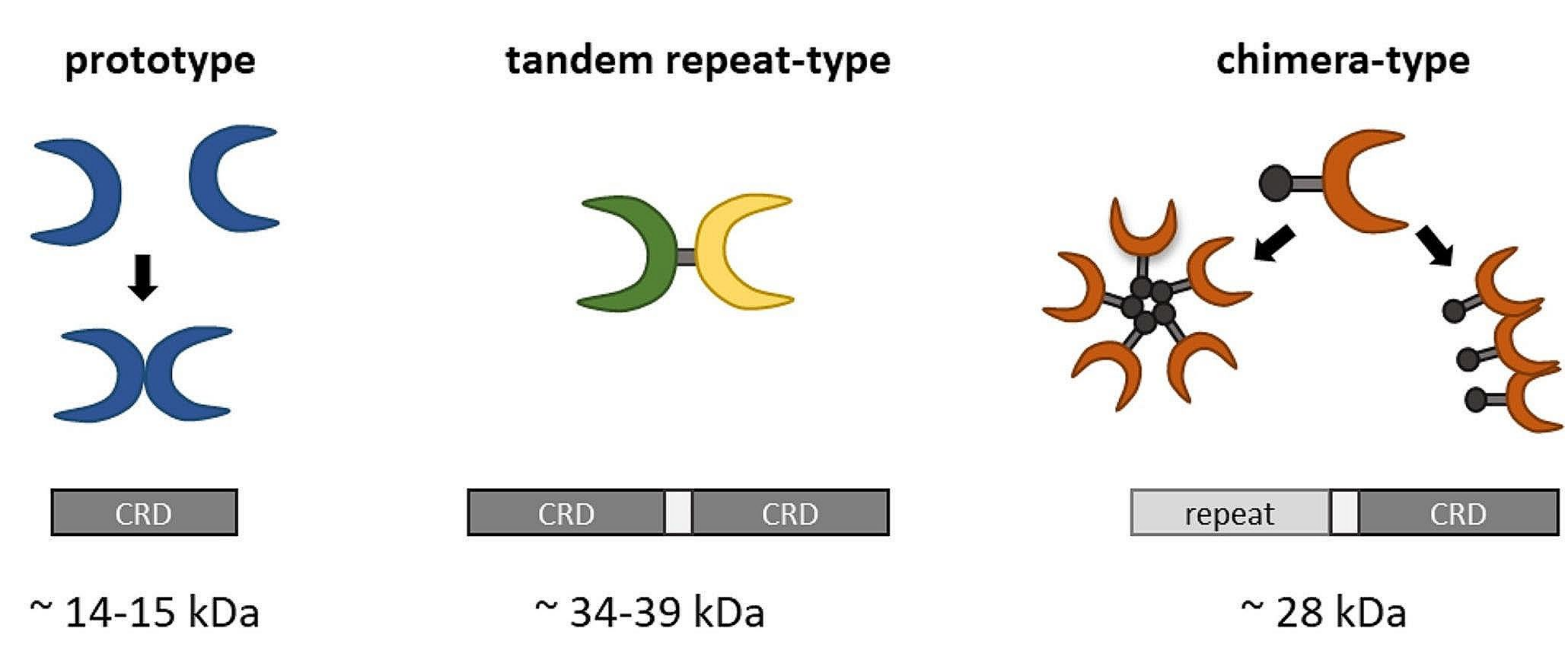
Domain structure of galectin subtypes. Prototype galectins contain one CRD, galectin-1, -2, -5, -7, -10, -11, -13, -14 and − 16. They are often characterized by a dimeric quaternary structure. Tandem repeat-type galectin-4, -6, -8, -9 and 12 are characterized by two CRDs. The only chimera-type galectin with a dynamic proline/tyrosine/glycine-repeat N-terminal domain and one C-terminal CRD is galectin-3. Type-C and Type-N-self-association by the C- or N-terminal domain into oligomeric assemblies have been described

The first galectin was discovered 1975 in the electric eel (*Electrophorus electricus*) and was named electrolectin [3]. The term galectin was introduced in 1994 [2]. So far 19 galectin subtypes have been identified. These lectins share distinctive primary amino acid sequences and are characterized by containing one or two carbohydrate recognition domains (CRD). Based on the CRD-number and allocation, galectins were classified into three groups. Prototype galectins contain only one CRD domain and can dimerize by non-covalent binding (galectin-1, 2, 5, 7, 10, 11, 13, 14 and 16) (Fig. 1). Tandem repeat-type galectins are equipped with two distinct CRDs with individual sugar-binding capacities, which are linked by a stretch of 5 to 50 amino acids (galectin-4, 6, 8, 9 and 12). The sole member of the chimera-type galectins is galectin-3. This individual variant contains one CRD that is linked to a non-lectin proline-rich N-terminal domain. Galectins found in the human genome were classified by the HUGO Gene Nomenclature Committee into galectin-1, -2, -3, -4, -7, -7B, -8, -9, -9B, 9 C, -10, -12, -13, -14, and − 16 (Gene group: Galectins (LGALS)).

The CRD contains all amino acid residues associated with the carbohydrate binding and is assembled from two extended antiparallel β-sheets folded into a β-sandwich structure [4]. Phylogenetic CRD-analysis based on genomic and mRNA data revealed that galectins with two CRDs emerged from an original bi-CRD galectin, which arose by tandem duplication of the gene encoding the ancestral mono-CRD galectin [5]. The β-sandwich CRD structure is widespread and many viral glycan-binding proteins contain a similar structural architecture so that it is likely that these viral proteins originate from host lectins [6]. Galectins specifically interact with N-acetyllactosamine (LN, Galβ1,3GlcNAc or Galβ1,4GlcNAc), a component present in various N- or O-linked glycans found on proteins throughout the cell. The concentrations of lectins required for half-maximal saturation of LN-binding, also known as the equilibrium dissociation constant (Kd), typically range from ten to several hundred µM. For example, galectin-1 exhibits a Kd of 21.5 µM [7], galectin-2 has a Kd of 654 µM [8], Kds for galectin-3 range from 27.9 to 93 µM [8, 9], full length galectin-4 has a Kd of 9.3 µM [8] and galectin-7 has a Kd within the range of 270–410 µM [9]. Their affinity for binding to oligosaccharides is higher compared to monosaccharides, and this affinity increases with the number of glycans [10]. In general, galectin binding affinities for most oligosaccharidic structures is relatively low. This can be compensated by galectin-oligomerization to form multivalent interactions with target molecules [11]. Some galectins have a preferential tissue specific localization like galectin-7 in the skin, galectin-10 in eosinophils/regulatory T cells, galectin-12 in adipose tissue [12] and galectin-13, 14 as well as 16 in the placenta [13, 14]. Other galectins like galectin-1 and galectin-3 show a more ubiquitous distribution.

Here, we primarily focus on the interactions of galectins with distinct polypeptides in intracellular compartments under normal and pathological conditions.

## Galectin interactions in the nucleus

**Fig. 2:**
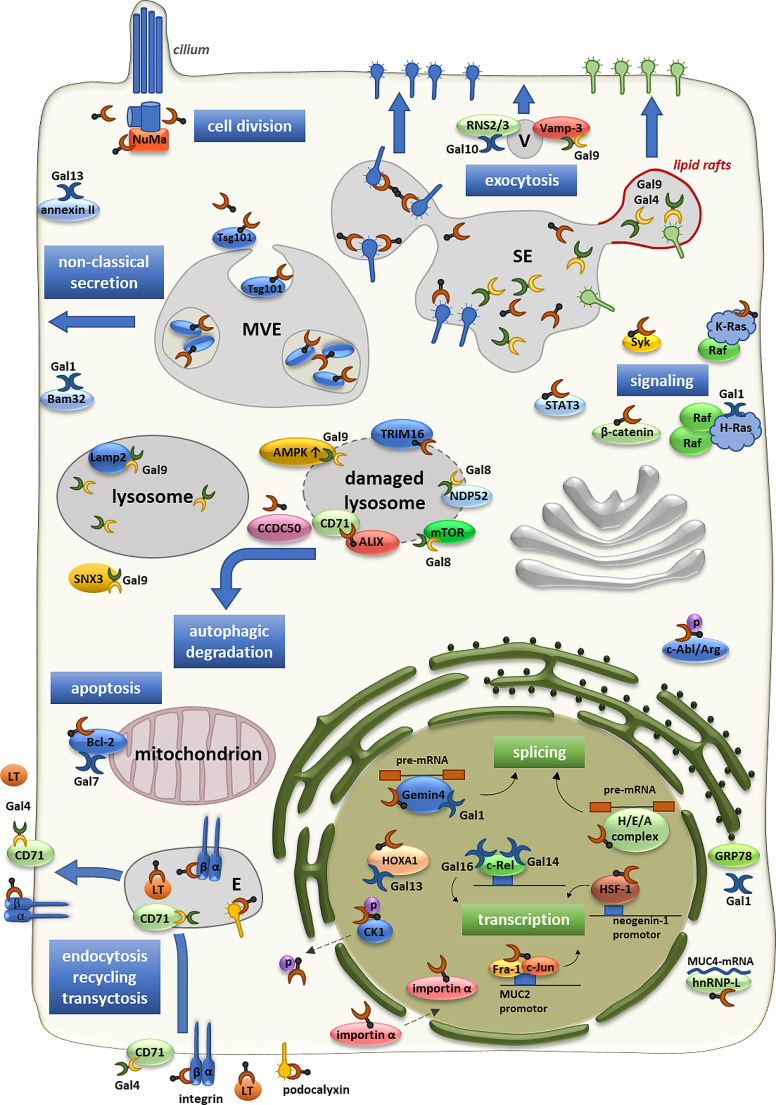
Intracellular binding partners of galectins in distinct cellular processes. Different cellular localizations and interaction partners of galectins are exemplified on a scheme depicting cellular organelles. Subtypes of prototype and tandem repeat galectins are indicated. E, endosome; LT, lactotransferrin; MVE, multivesicular endosome; SE, sorting endosome; V, transport vesicle

Some galectins possess classical nuclear localization sequences, resulting in their translocation from the cytosol into the nucleus (see Fig. 2). Examples include murine galectin-3 with the sequence (253)ITLT(256) [15] and human galectin-3 with (223)HRVKKL(228) [16]. The latter interacts with importin-α to facilitate passage through nuclear pores (refer to Table 1). Murine galectin-3’s N-terminus also demonstrates active nuclear transport capabilities, featuring a leucine-rich nuclear export signal (NES) at positions 241–249 [17]. Significantly, the phosphorylation of galectin-3 at Ser-6 by casein kinase 1 (CK1) appears to be involved in its nuclear export and anti-apoptotic activity, as illustrated in Fig. 2 [18, 19]. Phosphorylated galectin-3 is translocated from the nucleus to the cytosol, providing protection to the cells against drug-induced apoptosis. Consequently, the phosphorylation of galectin-3 at Serine-6 functions as a molecular switch, regulating its cellular translocation from the nucleus to the cytosol. Additionally, the pool of nuclear galectin-3 is augmented by a proline to histidine substitution at position 64, observed in breast and gastric cancers, thereby promoting cancer progression [20, 21].

**Table 1:** Intracellular galectin binding polypeptides

	nucleus	cytoplasm	vesicle lumen	
galectin-1	Gemin4 [24]	Bam32 [106]		
	TFII-I [30]	GRP78 [113]		
		H-Ras [44]		
		PCDH24 [120]		
		Raf effector [48]		
galectin-3	c-Jun, Fra-1 [34]	14-3-3σ [49]	CAECAM1, LPH, p75 [65]	
	CK1 [18]	ALIX [93]	DPPIV, NHE2 [71]	
	CRM1 [17]	bcl-2 [101]	integrin-β1 [73]	
	Gemin4 [24]	c-Abl/ Arg [124, 125]	lactotransferrin [77]	
	hnRNPA2B1 [23]	CCDC50 [99]	podocalyxin [75]	
	HSF-1 [35]	hnRNP-L [32]		
	importin-α [16]	K-Ras [46]		
	snRNPU1 [25]	NuMa [51]		
	Sp1 [36]	OGT [54]		
	STAT3/ β-catenin [129]	PCDH24 [120]		
	TFII-I [30]	Syk [109]		
		TRIM16 [97]		
		Tsg101 [57]		
galectin-4		SNX3 [84]	DPPIV, CEA, NCA, CD59 [69]	
		Src/ SHP2 [55]	lamp2 [80]	
			transferrin receptor [79]	
			vamp-3 [83]	
galectin-7		Bcl-2 [132]		
galectin-8		mTOR [134]		
		NDP52 [88]		
galectin-9		AMPK [134]		
		Rac1 [107]		
galectin-10		RNS2/ RNS3 [85]		
galectin-13	HOXA1 [38]	annexin II [39]		
galectin-14	c-Rel [42]			
galectin-16	c-Rel [41]			

Within the nucleus, galectin-3 is localized in nuclear speckles, as evidenced by its co-localization with the Ser-, Arg-rich splicing factor SC35 [22, 23]. These nuclear dots, in terms of both number and size, resemble two subnuclear domains known as Cajal bodies and gems (coiled Cajal bodies). Galectin-3, along with galectin-1, interacts with the gem marker protein Gemin4 (Fig. 2), as demonstrated through GST pulldown or immunoprecipitation experiments [24, 25]. Both of these galectins play roles in nuclear splicing of pre-mRNAs and gene expression [26, 27]. They have been shown to function redundantly as splicing factors for mRNA processing and nuclear export [28]. In human umbilical vein endothelial cells (HUVECs), the knockdown of galectin-1 has a notable impact on the expression of angiogenesis-related genes [29]. Through interactome and transcriptome analysis in this study, it was revealed that galectin-1 serves as a potent regulator of alternative splicing patterns for angiogenesis-associated genes, particularly those involved in focal adhesion and the angiogenesis-associated vascular endothelial growth factor signaling. The lectin may influence the transcriptome profiles of HUVECs by binding to RNA transcripts. Notably, using a galectin-1 mutant deficient in carbohydrate binding, Voss et al. demonstrated that carbohydrate binding is not implicated in the interaction of galectin-1 with spliceosomal components [30].

The sequence of events in galectin-interactions in spliceosome assembly has been studied in detail for galectin-3. When splicing is initiated, heterogeneous nuclear ribonucleoproteins (hnRNPs) assemble on the pre-mRNA to form the H complex (Fig. 2). One component of this complex is hnRNPA2B1, which interacts with galectin-3 [23]. If the N-terminal galectin-3-domain is added pre-mRNA splicing is arrested at a position corresponding to the H-complex, thus suggesting a functional role of galectin-3 in the first steps of splicing initiation [24]. Co-immunoprecipitation of galectin-3 from nuclear extracts also revealed components of the pre-spliceosome complexes E and A [23]. Evidence for interaction of galectin-3 with complex E came from earlier studies. This lectin fractionated in glycerol gradient separation studies from nuclear extracts in pre-mRNA particles together with the small nuclear ribonucleoprotein (snRNP) U1 thus forming a functional complex E [25, 31]. Galectin-3 itself is not a classical RNA-binding protein because it does not contain an RNA recognition motif [32]. Nevertheless, association of galectin-3 with snRNP U1 depends on the integrity of the snRNA of U1 and is affected in the presence of lactose. Together, these data strongly suggest that galectin-3 is involved in the initiation of spliceosome assembly and the splicing cascade.

In addition, mature mRNA can indirectly interact with galectin-3 in the perinuclear region. This has been demonstrated in pancreatic cancer cells, where the lectin binds to the hnRNP-L [32] (Fig. 2). It stabilizes the mRNA of the membrane-associated mucin MUC4, which activates genes involved in cell proliferation [33]. Expression of MUC2 is triggered by galectin-3 in intestinal cells by a different mechanism. Here, galectin-3 associates with c-Jun and Fra-1 in the nucleus to form an activating transcription factor complex at the AP-1 binding site on the MUC2 promoter [34]. Another example of galectin-3 as a transcription effector is provided by interaction with the HSF-1 transcription factor to promote expression of the cell adhesion molecule neogenin-1, cell survival in gastric cancer and cell motility [35](Fig. 2). Finally, involvement of galectin-3 in the cellular transcription machinery is demonstrated by its interaction with the transcription factor Sp1 in hematopoietic stem cells to upregulate p21 expression and control cell cycle progression [36].

Hox family proteins are transcription factors participating in embryonal development by gene expression regulation. Pull-down experiments revealed that placental galectin-13 binds to HOXA1, one of the earliest Hox genes to be expressed during embryonic development [37, 38]. HOXA1 can interact with the monomeric form of galectin-13 in the nucleus (Fig. 2). However, binding to dimeric galectin-13, which can be stabilized by disulphide bonds [39], is more efficient. It will be interesting to see future research on the consequences of this interaction on the cellular transcriptome especially in embryonic development. There is still another published nuclear galectin interaction that requires further characterization. With a sequence identity of about 60%, the two homologues prototype galectins galectin-14 and galectin-16 interact with the NF-κB family transcription factor c-Rel [40–42](Fig. 2). Consequences of this interaction may affect signal transduction, but the precise mechanistic details require further investigation.

## Cytosolic galectin interactions and non-classical galectin secretion

The functions of galectins are diverse and involve intricate interactions within cellular processes. In cellular signalling, galectin-1 is crucial for the nanoclustering of the small GTPase H-Ras [43, 44], while galectin-3 plays a similar role for K-Ras [45–47](Fig. 2). These nanoclusters, although short-lived, assemble on the cytoplasmic membrane leaflet, influencing signaling cascades. Galectin-1 specifically binds to and stabilizes dimers of the Raf effector, positively regulating GTP-H-Ras nanoscale signaling hubs [48]. A recent atomistic structural model of the Ras-Raf signalosome, including galectin-3 and 14-3-3σ, provides structural insight into these interactions [49]. In one scenario, galectin-3, capable of associating with the lipid bilayer [50], caps the farnesyl group of K-Ras in a hydrophobic binding pocket, facilitating the membrane localization of K-Ras assemblies. Notably, there is no evidence suggesting the involvement of sugar moieties in these cytosolic galectin functions. Conversely, galectin-3 associates with the nuclear mitotic apparatus protein (NuMA) in a sugar-dependent fashion [51] (Fig. 2). NuMA is a key mitotic regulator crucial for spindle pole establishment and cohesion. A mutant NuMA form blocked in O-GlcNAc-glycosylation leads to aberrant spindle pole formation. This mutant does not co-immunoprecipitate galectin-3, which suggests that the galectin-3-NuMA interaction depends on O-GlcNAc glycosylation. The exact mechanism remains unclear as O-GlcNAc does not bind to galectin-3 by itself. The cytosolic interaction of galectin-3 with basal bodies and centrosomes is significant, as depletion or knockdown of galectin-3 results in microtubule disorganization, perturbation of epithelial morphogenesis, and disorganization of primary cilia [52] or motile respiratory kinocilia [53].

It is interesting to note that in Hela cells galectin-3 can by itself be O-GlcNAcylated in the cytoplasm by O-GlcNAc transferase (OGT) [54]. This glycan modification, rarely found on secreted galectin-3 polypeptides, suggests that O-GlcNAcylation might trigger export of the lectin from the cytoplasm into the extracellular milieu. Additionally, tyrosine-phosphorylation by members of the Src kinase family affects the secretion of galectin-4 [55].

In general, secretion of galectins does not involve the classical transport pathway starting at the rough endoplasmic reticulum followed by vesicular transport to the Golgi apparatus, the *trans* Golgi network and endosomal compartments up to the plasma membrane. Instead, they are secreted by so-called non-classical mechanisms. One non-classical secretion scenario describes the sorting of cargo by members of the endosomal complex required for transport (ESCRT) into intraluminal vesicles (ILVs) at the membrane of multivesicular endosomes (MVEs) [56](Fig. 2). Galectin-3 interacts in the cytosol with Tsg101, a component of the ESCRT-I complex [57]. This direct interaction, facilitated by a highly conserved tetrapeptide P(S/T)AP motif (late domain motif) in the amino terminus of galectin-3, ensures recruitment into ILVs. The fusion of multivesicular endosomes (MVE) with the plasma membrane leads to the release of these vesicles as extracellular vesicles (EVs), functioning as communication platforms between cells or even organs within a living organism. The membrane protein E-cadherin is integrated into EV membranes through a comparable mechanism [58].

The primary secretion mechanism for galectin-3 from the apical membrane of polarized kidney epithelial cells is via EV-based secretion. Evidence for a functional role of cell-to-cell transfer of galectin-3-containing EVs comes from a study on the communication between B-cell precursor acute lymphoblastic leukemia cells and stromal cells [59]. Fei et al. discovered that galectin-3 released from stromal cells is internalized by target cells and transported into the cell nucleus. This process induces galectin-3 expression and provides protection to the target cells against drug treatment.

While a late domain motif has not been identified in other galectin-family members, the presence of galectin-1 [60], galectin-4 [61], galectin-5 [62] galectin-9 [61] and galectin-13 [63] in extracellular vesicle pools suggests recruitment by yet unidentified mechanisms. Interaction of galectin-13 with cytosolic annexin II at the plasma membrane may play functional roles in the non-classical secretion of this lectin from placenta and fetal hepatic cells [39](Fig. 2). Alternative pathways for non-classical secretion of galectins are also under discussion. The interaction capacity of galectin-3 with membrane lipids suggests the possibility of spontaneous penetration of lipid bilayers [50]. The whole matter of non-classical secretion of galectins has not been settled yet and further studies are required to fully understand all processes involved. Notably, galectin-10 is released by eosinophil cells through a process of active cell death referred to as ETosis, elevating serum levels of galectin-10 [64]. This lytic degranulation of eosinophile cells elevates serum levels of galectin-10.

## Galectin binding partners in endosomal, lysosomal or vesicular lumina

Galectins can be secreted and endocytosed or recruited into MVEs to reside within endosomes, lysosomes or transport vesicles, where they function as mechanistic components of vesicular trafficking. Internalized galectin-3, -4 and − 9 are involved in the apical endocytic-recycling of newly synthesized glycoproteins [65–69](Fig. 2). While being transported from the *trans* Golgi network (TGN) to the plasma membrane, galectin-3 binds to glycans that are exposed on the neurotrophin receptor. This binding leads to the clustering of the neurotrophin receptor, a non-raft-associated glycoprotein, into high molecular weight clusters. These clusters are then recruited into apical transport vesicles [70]. Galectin-3 is involved in apical targeting of the carcinoembryonic antigen-related cell adhesion molecule 1 (CAECAM1), dipeptidylpeptidase IV (DPPIV), lactase-phlorizin hydrolase (LPH), the neurotrophin receptor (p75), sodium-proton exchanger 2 (NHE2) and integrin-β1 [71–73]. A distinct set of lipid raft-associated apical cargo molecules is sorted by galectin-4 into apical transport carriers [66, 69]. In addition to binding to glycoproteins, the two carbohydrate recognition domains (CRDs) of galectin-4 interact specifically and with high affinity with a defined pool of glycosphingolipids. Specifically, this interaction involves sulfatides with long fatty acid chains, hydroxylated in position-2. The binding of galectin-4 to raft constituents results in cross-linking, forming raft clusters. These clusters are postulated to contribute to the formation of membrane microdomains that subsequently bud off to generate apical transport carriers. Once at the apical membrane, galectins can undergo re-internalization. It’s worth noting that the efficiency of galectin-3 internalization is pH-dependent and can be competitively inhibited by lactose [74]. Recent evidence indicates that the sialomucin podocalyxin establishes a ligand-receptor pair with galectin-3, initiating the co-internalization of both molecules from the apical membrane [75](Fig. 2). The endocytic process involves galectin-3-mediated cargo clustering and membrane bending, playing a functional role that is dependent on glycosphingolipids, as demonstrated in prior studies [76]. In mouse enterocytes, a glycosphingolipid-dependent internalization of lactoferrin and galectin-3 from the apical membrane has been previously described [77]. It also seems plausible that vesicular recycling of galectin-3 between the plasma membrane and endosomal organelles is maintained by alterations in the interaction partners. This view is supported by pH-dependent fine-tuning of galectin-3 cluster formation [78]. Moreover, the interaction between galectin-3 and integrin-β1, as well as galectin-4 with the transferrin receptor, facilitates basolateral to apical epithelial transcytosis [73, 79](Fig. 2). The elimination of these interactions results in the lysosomal targeting of the associated cargo molecules. This observation suggests that galectin-mediated apical sorting is not limited to newly synthesized cargo molecules but extends to previously existing ones as well.

Galectin-9 is enriched in the lumen of lysosomes from enterocytes and predominantly binds to the lysosome-associated membrane protein 2 (Lamp2) [80](Fig. 2). This lectin interaction, largely with the N-glycan chain attached to Asn-175 of Lamp2, stabilizes lysosomes, maintains lysosomal acidity, and facilitates lysosome-mediated autophagy to prevent ER stress and associated cell apoptosis in intestinal Paneth cells and acinar cells of the pancreas. In dendritic cells, mast cells and macrophages galectin-9 is involved in cytokine secretion [81–83]. Intracellular galectin-9 interaction with the soluble NSF attachment receptor vesicle associated membrane protein 3 (VAMP3) regulates cytokine secretion in dendritic cells [83](Fig. 2). Interestingly, galectin-9 binds to the C-terminus of cytosolic sorting nexin-3 (SNX3) in macrophages to regulate compaction of intact *Borrelia*-containing phagosomes, thus driving their maturation and *Borrelia* degradation [84]. However, the exact role of galectin-9 in the curvature-sensitive process of *Borrelia* phagosome compaction is not known yet.

The Charcot-Leyden crystal component galectin-10 interacts with glycosylated human eosinophil granule cationic RNAses, namely, eosinophil-derived neurotoxin (RNS2) and eosinophil cationic protein (RNS3), in a glycan-independent fashion [85]. Lectin-interaction does not inhibit the endoRNAses, but may play a role in their vesicular transport from granulogenesis until secretion in mature eosinophile cells.

## Galectin binding involved in lysosomal damage

Recent evidence points on the involvement of galectins in lysosomal homeostasis. Lysosomes can be damaged by exposure to biologically active silica crystals, proteopathic fibrils or microbial invasion. Once damaged lysosomes are beyond repair, they are removed by autophagy (for review see [86]). In this process galectins in conjunction with ubiquitination serve as “eat me” signals to control orchestration of several autophagy regulators. They recognize membrane damage by binding to lumenal β-galactosides once glycoconjugates on the exofacial leaflet are exposed to the cytosol and bind to and recruit autophagic receptors. Initial publications of this phenomenon describe binding of galectin-3 to host glycans exposed on lysed vacuoles following *Shigella*, *Listeria* and *Salmonella* infection [87]. Thereafter, it was shown that galectin-3, -8 and − 9 sense the exposure of host glycans on membranes ruptured by *Listeria*, *Salmonella* and *Shigella* infection [88]. Mechanistic insight was provided by the finding that binding of the autophagic receptor NDP52 to the C-terminal CRD of galectin-8 and ubiquitin targets damaged *Salmonella*-containing vacuoles and activates antibacterial autophagy (Fig. 2). Moreover, in response to lysosomal damage galectin-8 dynamically associates with and inactivates the Ser/Thr protein kinase mTOR, which acts as a negative regulator by phosphorylating inhibitory sites on regulators of autophagy [89]. In conjunction with proximity biotinylation proteomics data these observations indicate that galectin-8 is a key regulatory node for lysophagy [90]. Galectin-9, which is also important for an optimal autophagic response, activates the AMP-activated protein kinase (AMPK) by displacement of the deubiquitinase USP9X from AMPK-TAK1-complexes to promote ubiquitination of TAK1 [89, 91]. The sequence of events in the action of galectin-3 on damaged lysosomes starts with targeting of the lectin to damage-exposed glycans. Here, interaction of the lectin with glycans exposed on the transferrin receptor (CD71) is functionally important for Gal3 recruitment to damaged lysosomes [92](Fig. 2). Galectin-3 then interacts with the ESCRT component ALG-2-interacting protein X (ALIX) [92–94] to facilitate ESCRT-III complex formation and mediate lysosomal repair [95, 96]. The intriguing aspect is that the interaction between galectin-3 and ALIX intensifies as lysosomal damage progresses. Simultaneously, the interaction with the ESCRT-I component Tsg101 decreases. This observation suggests a scenario in which ALIX selectively interacts with galectin-3 to facilitate lysosomal repair, while the association with Tsg101 aids in recruiting the lectin into multivesicular endosomes (MVEs) for extracellular vesicle (EV) secretion. At a later step, when damaged lysosomes have to be removed by autophagy, galectin-3 switches from ALIX to the tripartite motif protein TRIM16 [97]. Here, the CRD-domain of galectin-3 interacts with TRIM16 to recognise endomembrane damage and associate with the key autophagy regulators ATG16L1, ULK1 and Beclin 1. They govern cargo degradation in a highly precise process [98]. Very recently, interaction of galectin-3 with the lysophagy receptor coiled-coil domain-containing 50 (CCDC50) has been demonstrated to control lysosomal integrity and renewal [99]. This interaction may not be direct. In melanoma CCDC50 is upregulated and the galectin-3/CCDC50-mediated lysophagy mechanism supports tumour growth and, potentially, also works in other tumours. Thus, especially within the last decade many aspects in the complex network of galectin interaction partners in lysosomal damage and autophagy have been uncovered to yield a cohesive view of galectin activities in this field.

## Intracellular galectin interactions in immune cells

Extracellular galectin-1 and galectin-3 are both inducing T cell apoptosis by interaction with glycoprotein receptors CD45 and CD71 on the cell surface, which weakens the T cell mediated immune response [100]. It is thus surprising that intracellular galectin-3 has the opposite effect on T cell apoptosis. In Jurkat cells the carbohydrate binding domain of galectin-3 intracellularly interacts with the apoptosis inhibitor Bcl-2 to suppress apoptosis and promote cell growth [101]. Furthermore, Mohammadpour et al. demonstrated that the expression of galectin-3 provides partial protection to T cells against apoptosis induced by IFN-γ [102]. It remains unclear whether this protection is contingent upon extracellular or intracellular interactions of galectin-3. Nevertheless, antiapoptotic galectin-3 function is consistent with its known anti-apoptotic role in other cell types, including cancer cells [103]. The study of Mohammadpour et al. also shows that galectin-3 acts as a negative regulator of T cell function and proliferation [102]. The conflicting impacts, with its antiapoptotic properties on one side and the dampening of the immune response on the other, highlight the nuanced role of galectin-3 in T cells. A documented attenuation of the immune response by intracellular galectin-3 has specifically been observed in activated T cells. Here, galectin-3 interaction with the ESCRT component ALIX is focussed to the stable contact region between T cells and antigen-presenting cells (APCs) called the immunological synapse (IS) [93]. This cytoplasmic interaction may promote T cell receptor downregulation and T cell inactivation by attenuation of ALIX-mediated trafficking events. In addition, Kaur et al. describe an increase in intracellular galectin-3 upon activation of CD8^+^ T cells following herpesvirus infection in comparison to the secreted galectin-pool [104]. In activated CD8^+^ T cells, cytoplasmic galectin-3 is mobilized to the immunological synapse (IS), where it diminishes both their proliferation and cytokine production, thereby impeding T cell functions. In contrast, galectin-9 exerts a positive influence on the immune response. It is recruited intracellularly to the IS upon T cell activation and promotes T cell receptor signalling, which affects T cell differentiation and B cell responses [105]. The precise mechanism of this galectin-9 mediated function, however, has not been clarified yet. Dendritic cells express galectin-1 which intracellularly interacts with the cytoplasmic adaptor protein Bam32 once the cell has reached the mature state [106](Fig. 2). This interaction might modulate the stimulatory capacity of mature dendritic cells to activate CD8^+^ T cells. Moreover, galectin-9 is also expressed in dendritic cells and has been shown to organize the cortical actin cytoskeleton to optimize their phagocytic capacity [107]. Knockdown experiments suggest that the underlying mechanism is based on a galectin-9 mediated promotion of Rac1-GTP activation, which then reorganizes the actin cytoskeleton and affects membrane rigidity.

Intracellular interactions of galectin-3 also suppress immune responses in neutrophil and dendritic cells. In neutrophil cells galectin-3 inhibits the neutrophil ROS response to phorbol myristate acetate or zymosan stimulation and regulates the life span of neutrophils during virulent *Toxoplasma* infection [108]. Importantly, mechanistic insight into the role of galectin-3 in attenuation of neutrophil cells came from a subsequent study on phagocytosis of *Candida* [109]. Wu et al. found that galectin-3 physically interacts with the tyrosine kinase Syk (Fig. 2), which suppresses complement receptor 3 (CR3) downstream Syk-mediated ROS production and anti-*Candida* function of neutrophil cells. Another study with dendritic cells did not observe galectin-3 mediated effects on Syk-induced signalling after stimulation of dectin-1 or TLR4, two different classes of pattern recognition receptors that identify molecular components expressed on microbial pathogens [110]. Instead, they demonstrate that galectin-3 affects cytokine expression of dendritic cells to suppress T cell responses. These observations are in line with data from *Leishmania* infected knockout mice showing that galectin-3 deficiency leads to increased activation of the Notch signalling pathway, and enhanced production of both pro- and anti-inflammatory cytokines [111]. Some of the observed effects in these studies can be explained by extracellular interaction of galectin-3 with glycan moieties exposed on the extracellular receptor domains. Nevertheless, the finding that neither the presence of lactose nor the addition of recombinant galectin-3 to dendritic cell cultures affected cytokine production also suggests a contribution of intracellular galectin-3 mediated interactions.

Overall, these studies indicate that depending on the galectin-variant or interaction partners cytosolic association with galectins provides ambivalent characteristics that can either dampen or elevate the immune response.

## Intracellular binding partners of galectins in cancer

Many of the described roles of galectins in cancer can be assigned to interactions in the extracellular milieu. Nevertheless, some examples show that intracellular galectin interactions determine the fate of cancer progression. Cytosolic interaction of galectin-1 with H-Ras as mentioned above, stabilizes anchorage of the complex on the cytoplasmic membrane leaflet and further activates downstream oncogenic pathways together with the Raf effector [112](Fig. 2). This oncogenic role of galectin-1 corresponds to the poor survival rate of patients with a high expression pattern of this galectin subtype in gastric cancer [113], lung cancer [114], renal cancer [115], hepatocellular carcinoma [116] and ovarian cancer [117]. Moreover, in the cytosol and the nucleus galectin-1 colocalizes with the glucose-regulated protein 78 (GRP78), most likely the cytosolic variant of this ER-chaperone [113](Fig. 2). GRP78, which is also known as BIP, is overexpressed in various cancer cells and contributes to tumour cell survival [118]. Molecular details of this interaction are only vaguely understood. Clues might come from the observation that GRP78 is closely related to and promotes epithelial–mesenchymal transition (EMT) in cancer cells [119], which is also affected by protocadherin-24 (PCDH24), another galectin-1 interacting polypeptide [120, 121].

In analogy to galectin-1, galectin-3 interaction with K-Ras mediates membrane localization, stabilizes the GTPase in its active state and presents composite interfaces to facilitate Raf binding [49, 122, 45–47]. The multifaceted roles of galectin-3 in cancer progression have been summarized by Thijjsen et al. [123]. Of notice, galectin-3 itself is posttranslationally modified in distinct cancer tissues. In human breast cancer cells, the non-receptor tyrosine kinases c-Abl/Arg directly interact with and phosphorylate the N-terminal domain of cytosolic galectin-3 (Fig. 2). This phosphorylation occurs at tyrosine residues 79, 107, and 118, with Tyr-107 identified as the primary phosphorylation site [124, 125]. Moreover, galectin-3 can undergo cleavage, likely extracellularly, at this site by the chymotrypsin-like serine protease prostate-specific antigen (PSA) [126]. The phosphorylation at Tyr-107, mediated by c-Abl, inhibits this cleavage process. Consequently, unphosphorylated galectin-3 secreted by non-cancerous prostate cells is susceptible to cleavage by PSA, leading to the removal of the N-terminal domain. This cleavage event has the potential to reduce oligomerization and lattice formation by galectin-3 [127]. Interestingly, phosphorylation also seems to determine the intracellular fate of galectin-3 in cancer cells. If c-Abl/Arg kinases are absent, intracellular galectin-3 is recruited into lysosomes and degraded [125].

As mentioned before, the serine/threonine kinase CK1 phosphorylates galectin-3 at Ser-6 (Fig. 2). Apart from modulation of the subcellular lectin distribution between the nucleus and the cytosol, this phosphorylation is also required for tumor necrosis factor-related apoptosis-inducing ligand (TRAIL)-induced apoptosis of breast cancer cells [128]. Phosphorylated galectin-3 activates a non-classical caspase activation cascade through induction of PTEN expression and inactivation of the PI3K/Akt survival pathway. The precise mechanisms responsible for galectin-3-mediated PTEN expression remain unclear at present. However, it appears that these mechanisms are facilitated by nuclear interactions of the lectin with transcription factors and/or heterogeneous nuclear ribonucleoproteins (hnRNPs). The possibility of an interaction between phosphorylated galectin-3 and PTEN cannot be ruled out either.

A different site of action for galectin-3 are the WNT/β-catenin and STAT3 signalling pathways (Fig. 2). Binding of galectin-3 to the transcription factor STAT3 enhances its Tyr-705 phosphorylation, leading to its nuclear translocation and transcriptional activation in gastric cancer tissues [129]. Furthermore, galectin-3 expression is elevated by WNT/β-catenin signalling. The lectin also interacts with β-catenin and promotes its translocation into the nucleus for activation of target genes. This study suggests that galectin-3 mediates the crosstalk between WNT/β-catenin and STAT3 signalling in tumour progression. Consequently, increased expression and co-localization of β-catenin, pSTAT3, and galectin-3 in patients with advanced gastric cancer correlates with a poorer prognosis.

Intracellular interaction of galectin-13 with the transcription factor HOXA1 does not seem to be exclusively important for embryonic development but also appears to be involved in cancer progression, since upregulation of HOXA1 is associated with a poor prognosis in patients with breast cancer or hepatocellular carcinoma [130, 131].

For galectin-7, interaction with mitochondrial Bcl-2 is implicated in the regulation of apoptotic cell death of colon carcinoma cells [132]. Bcl-2, which resides in the outer mitochondrial membrane oriented towards the cytosol, directly interacts with galectin-7 and recruits the lectin independently of carbohydrate recognition to mitochondria (Fig. 2). Mitochondrial localization and intracellular functioning of galectin-7 in apoptosis had been previously reported [133]. Upon binding to Bcl-2 galectin-7 may act as a pro-apoptotic protein that assists in initiation of the apoptotic cascade in cancer cells.

## Concluding remarks and future perspectives

The past three decades have witnessed a significant increase in the identified intracellular interaction partners of galectins. Obviously, the focus of many of these studies concentrates on prototype galectin-1 and chimeric galectin-3. This is most likely due to the ubiquitous expression of these two galectins in many distinct tissues. Nevertheless, even if we narrow down our attention on these two galectins, many of the cellular interactions and their consequences still require further elucidation, especially on the molecular level. With respect to the remaining members of the galectin family extensive analysis of their intracellular roles should not be neglected. Even if just a defined number of examples is discussed in this review, it is self-evident that each member of this family possesses its individual pattern of cytosolic binding partners that await to be identified. We will have to fully characterize this network and identify the molecular details to develop strategies to cure diseases associated with galectin functions.

## Data Availability

Data sharing is not applicable as no datasets were generated or analysed for this review article.
